# The benefits of online access to prescription medicines: the European patient’s perspective

**DOI:** 10.3389/fpubh.2025.1513338

**Published:** 2025-09-08

**Authors:** Christian Jervelund, Bruno Basalisco, Malwina Mejer, Marco Islam, Mads Thorkild Nissen, Michael Isles

**Affiliations:** ^1^Copenhagen Economics, Copenhagen, Denmark; ^2^Copenhagen Economics, Brussels, Belgium; ^3^Alliance for Safe Online Pharmacy in the EU, Essex, United Kingdom

**Keywords:** prescription, online, chronic, convenience, adherence

## Abstract

**Background:**

In the evolving EU healthcare landscape, online access to prescription medicines is rising. The EU legislation provides a legal framework for safe online access, aligning supply rules with physical pharmacies and providing specific measures against counterfeiting. Eight Member States allow online access to prescription medicines.

**Objectives:**

The study aims to understand factors influencing EU citizens’ propensity to obtain prescription medicines online.

**Methods:**

The online survey was conducted in Germany, France, Italy, Spain, and Sweden with 1,000 responses per country, to help ensure representatives in terms of age, gender, and macro-region. Parametric tests and regression analysis to control for confounding factors were used. In countries where online access to prescription medicines was allowed, current usage choices and preferences was asked. In countries where online access was not allowed, questions about the intended use and preferences if prescription medicines became available online was asked.

**Results:**

Thirty four percent (34%) of respondents expressed interest in obtaining prescription medicines online. This interest is was slightly higher among people with chronic conditions (36.2%) and their caregivers (41.4%). Respondents facing long distances to pharmacies or perceiving their opening hours as inconvenient expressed a preference for online access. Additionally, a number of respondents believed that online access could improve adherence to prescribed courses of medication. In particular, 64% of the chronically ill respondents who reported adherence problems due to time constraints believed that online access would improve their adherence. In countries where online access was permitted, rates of non-adherence caused by time constraints were 22 percentage points (*p* < 0.001: 95%CI: 0.153–0.298) lower. An assumption might be therefore made that online access could benefit both caregivers and chronically ill patients, by managing adherence more effectively.

**Conclusion:**

Given the findings in this large scale survey and taking in to account the pharmacists interviewed about potential practical barriers, it would seem reasonable for EU Member States who currently prohibit online access to prescription medicines to consider reviewing the current national legislation.

## 1 Introduction

There is currently a focus in the European Union (EU) on digital technologies which as the potential to enhance healthcare. The introduction of e-prescriptions (this is where a healthcare professional who is authorized to write a prescription, does so via an electronic digital software package) allow medical practitioners to issue precise, error-free, and easily understandable prescriptions stored electronically for access by pharmacies or patients. The patients will be asked where they would like the prescription to be dispensed, i.e., a choice of a local pharmacy or the patient may decide to obtain the dispensing of their prescription using an online pharmacy.^1^ Studies back up the desirability of accessing prescription medicines online. In one study carried out in Poland, France and Spain with a sample size of 1,500 patients (average age of 41.1 years) 90% of respondents said that on-line pharmacies should be permitted to deliver prescription medicines. And 85% of them would buy prescription medicines online if allowed.^2^ The recent introduction of the European Health Data Space (EHDS) emphasizes the political will to enhance the population’s health through digital solutions. This is described by the European Commission as a cornerstone of the European Health Union and the first common EU data space dedicated to a specific sector as part of the European strategy for data. The EHDS Regulation aims to establish a common framework for the use and exchange of electronic health data across the EU. It enhances individuals’ access to and control over their personal electronic health data, while also enabling certain data to be reused for public interest, policy support, and scientific research purposes. It fosters a health-specific data environment that supports a single market for digital health services and products. Additionally, the regulation establishes a harmonized legal and technical framework for electronic health record (EHR) systems, fostering interoperability, innovation, and the smooth functioning of the internal market. In this context it is encouraging to note that a recent study carried out in Germany showed that Online pharmacies have established themselves as a firm pillar in the supply of medicines and recorded an 80% increase in orders placed with online pharmacies between 2019 and 2023^3^.

Such an infrastructure enhances the possibilities of enabling digital solutions which may include the convenience gains by obtaining prescriptions online, enabling individuals to access their required medications from the comfort of their homes. This transition should streamline the prescription process and has the potential to enhance accessibility, making healthcare more adaptable to the diverse medical needs of patient groups. A clear example of this is the NHS App which allows access to NHS services such as ordering repeat prescriptions, a GP health record, upcoming and past appointments and checking if you need urgent medical help using 111 online.^4^

It is important to note that the increase in falsified medicines via online websites needs to be addressed^5^^,^^6^^,^^7^^,^^8^^,^^9^. Many people are not aware that websites are selling falsified medicines^10^ and patient safety organizations involved in promoting online safety believe the sooner all countries allow legal and regulated services, the sooner the public will benefit^11^. The conclusion of a large meta-analysis which systematically reviewed online pharmacies selling prescription medicines concluded that stricter regulation by the World Health Organisation is needed and it can be argued where a country does not allow the legal transaction of a prescription medicine online, it opens itself up to forestalling an increased awareness of where to obtain prescription medicines online safely.

While the benefits of e-prescriptions are well researched and documented, much less is known about the potential benefits of online access to prescription medicines. Implementing e-prescriptions enhances efficiency, minimizes medication errors, and improves convenience [e.g., (1–3)]. The benefits of online access, however, are limited to the convenience benefits of e-commerce, such as the ability to order at any time and save time [e.g., (4, 14, 16, 18, 19)]. This is surprising given the potential of innovative, digital solutions to address long-lasting problems of non-adherence to medication (5).

This research aims to understand how European citizens perceive the option of accessing their prescription medicine online and the impact of such access on adherence to medication.

This study is the first to systematically analyze the demand for online access to prescription medicines in Europe.

Despite the growing interest in online access to prescription medicines, several barriers hinder patient access and utilization. Studies point to regulatory challenges, technological limitations, counterfeiting risks, lack of awareness, and privacy concerns as key obstacles preventing patients from fully benefiting from online access^12^. Within the European Union, existing regulations provide access safeguards. First, the Falsified Medicines Directive^13^ introduced safety features, such as unique identifiers: each prescription pack with a data matrix bar code (which must be digitally decommissioned at the point of dispensing for authenticity by a scanner device) coupled with a pack that has a tamper evidence seal. Moreover, it obliged all pharmacies selling medicines via the Internet to display EU Common Logo, with the pharmacy address and license linked to a national register, enabling patients to authenticate the website with a simple click^14^. The regulatory framework further requires that online pharmacies must follow the same secure supply chains as offline physical pharmacies^15^. The upcoming Digital Identity Wallet^16^ will facilitate secure, protected access to public and private online services across Europe.

Barriers related to the lack of awareness and constraints on pharmacy owners to adopt an online prescription service for their patients are also explored in this study.

This article is structured as follows. Section 2 outlines the methodology, while Section 3 discusses the results concerning any perceived convenience and health benefits of online access to prescription medicines. Section 4 highlights key barriers to the broader adoption of online solutions, focusing on the lack of awareness, technological limitations, and constraints on the digital expansion by pharmacies wishing to potentially provide more services. Section 5 discusses limitations, and Section 6 provides the conclusion.

## 2 Methodology

### 2.1 Survey

The survey methodology was developed and designed by the authors of this manuscript combined with the input from industry experts. The research methodology employed in this study centered around a large-scale survey (see Appendix 8.1) administered through a market research company in the fall of 2023. Data was collected from participants across five EU Member States: France, Germany, Italy, Spain and Sweden. The following details outline the methodological approach of the survey. The focus was on individuals who have procured goods and services through online channels and who obtained prescription medicine in the past year and on chronic patients. Informed consent was obtained from all survey participants by the market research company prior to their participation in the study. The reasons for this choice of sampling is as follows (a) the research wanted to measure, if users already acquainted with e-commerce have an interest for accessing e-shopping of prescription medicines in a safely regulated way; (b) to fit within the study budget, an online survey was necessary and this is by its nature was directed to respondents who have and use online tools; (c) online survey allows the study to address potential social desirability bias in replies. Whilst a broader sample could have been included to those who had not procured goods and services online, the budget did not permit this.

Sample Size and Distribution: Five thousand (5,000) respondents were recruited to ensure a comprehensive data set and strategically distributed the sample, selecting 1,000 respondents per country. The study was designed intentionally to choose countries representing larger populations and those with differing regulations regarding online access to medicines. Specifically, Germany and Sweden were included, where the online purchase of prescription medication is permitted, and contrasted these with France, Italy, and Spain, where such online prescription medicines is not possible. This approach allows a juxtaposition of expectations and actual experiences of EU citizens regarding online access to prescription medicines.

Demographics: The survey sample ensured that participants were representative in terms of gender, age^17^, and macro-region in each of the five EU countries. However, an overrepresentation of individuals with a chronic condition was introduced, given the significance of their situation to research the effects of online access to prescription medicines^18^. The survey was administered via an online panel (also due to budget reasons) and thus all respondents were by design connected to the internet and familiar with online tools. Furthermore, the sample choice ensured that respondents had a basic familiarity with e-commerce, having shopped online (any product/service) within the last 12 months. These features are inline with the majority of people in the EU, namely 64% of EU residents^19^. It is a fact that internet access is greater in urban areas^20^ and that the propensity to shop online is higher among individuals up to 55 years old^21^; therefore, our sample is likely overrepresenting these groups.

Survey Design: The survey questions were designed to assess respondents’ experiences, preferences, and attitudes toward online access to prescription medicines. In countries where online access to prescription medicines is allowed, questions were asked about current usage choices and preferences. In countries where online access is not allowed, questions about intended use and preferences if prescription medicines became available online were asked. Importantly, all questions were translated into the respective language of the country and tested each version with native speakers before their launch to ensure that all questions were clear and concise.

The primary focus of our survey was to explore the factors influencing people’s propensity to order medicine online. There was a focus on individuals with access to the internet and basic familiarity with online use. The study aimed to understand whether providing online access enhances convenience in obtaining prescription medicines and whether it can potentially improve their adherence to regular medication regimes. In addition to these focal points, a variety of other questions were incorporated to deepen our understanding of online access to prescription medicines. This broader approach was designed to uncover more comprehensive insights into the needs and demands of society.

Relying on self-reported data raises concerns about potential biases, such as social desirability bias and recall bias. The study tried to address this bias within the study design as follows. Social desirability bias occurs when participants respond in a way that they believe is expected of them [see, e.g., (6)]. Because medication adherence is considered a socially desirable behavior, respondents may underreport instances of non-adherence. An online survey that ensures anonymous responses helps to reduce such bias (7). Recall bias arises from the ability of participants to accurately recall past behaviors. To reduce this bias, time-bound questions were used (8). Specifically, for medication dispensing, questions were posed about the most recent experience of obtaining prescription medicine, and for adherence, questions were asked about experiences during the last 6 months. Time-bound questions help respondents remember specific details rather than relying on generalizations or inferences, thus reducing recall bias.

### 2.2 Statistical analysis

Many of the results are directly derived from the survey responses and can be effectively described using summary statistics. However, inferential statistics are used to detect relations and correlations in the data and examine the effects of different influencing factors. In the analysis, parametric tests are employed and a linear regression framework, which enables control for confounding factors. This approach allows us to identify a clear effect when investigating correlational or causal relationships within the data.

In the analysis, a significant portion of the data is captured using a five-point Likert scale. The challenge with Likert scales is that their ordinal nature can complicate the interpretation and comparison of variables due to their reliance on subjective assessments. To address this issue, the variables were standardized, thereby facilitating a simpler interpretation and comparison. The standardization process transforms the Likert scale variables and allows an interpretation of the results in terms of standard deviations (9). Additionally, when performing regression analysis, robust standard errors to account for potential heteroskedasticity were used, ensuring the reliability of our inferences even in the presence of non-constant error variance across observations (10, 11).

## 3 Results

Of the 5,000 respondents who participated in our survey across the five EU countries, 34% indicated that they are or would be “likely” or “very likely” to order prescription medicine online. The share is highest in Sweden (53%) where online access is already available and a well-established service and lowest in France (21%). See Table 1.

**Table 1:** How likely are you to order a prescription medicine online?

	Sweden		Spain		Italy		Germany		France	
	*n*	%	*n*	%	*n*	%	*n*	%	*n*	%
Very unlikely 1	133	13.3	289	28.9	161	16.1	341	34.1	341	34.1
2	118	11.8	142	14.2	109	10.9	169	16.9	169	16.9
3	223	22.3	261	26.1	313	31.3	264	26.4	264	26.4
4	150	15.0	173	17.3	176	17.6	90	9	90	9
Very likely 5	376	37.6	135	13.5	241	24.1	136	13.6	136	13.6

In the remainder of this section, the descriptive statistics are summarized (Tables 2–5) and explore the drivers behind what are the motivators for online access. The focus is on four groups that could benefit the most: chronically ill people and their caregivers due to the high burden of regular pharmacy visits, individuals who face long distances to pharmacies, and those who perceive their opening hours as inconvenient. In conducting this analysis, we employ gender, age, and income as control variables. Controls for education level, country effects, city size, and the respondent’s general propensity to shop online (i.e., products other than medicines) were added.

**Table 2:** Showing numbers with and without a chronic disease.

Country	Those with a chronic disease	Those without a chronic disease	Prefer not to say
Sweden	244	743	13
Spain	488	506	6
Italy	500	481	19
Germany	712	275	13
France	737	351	11
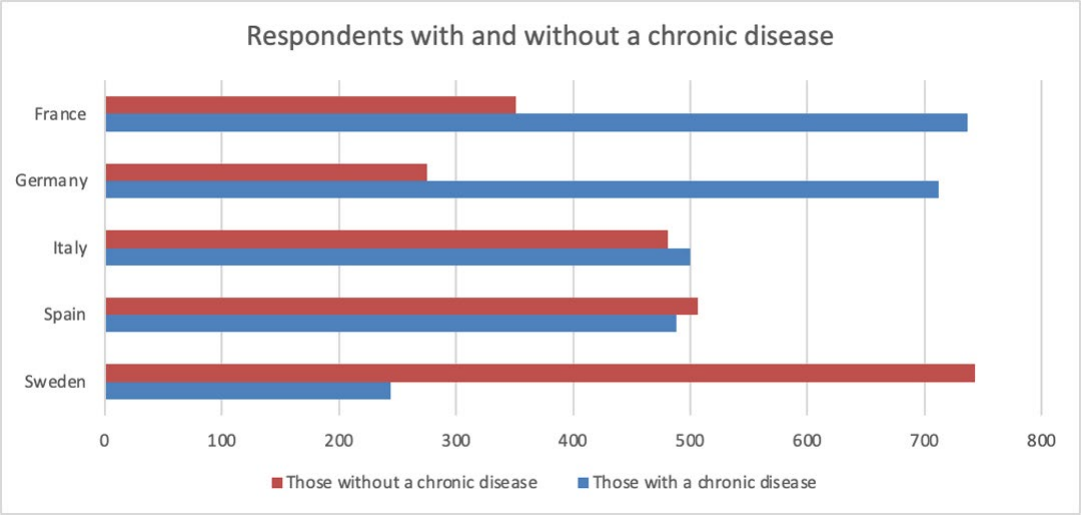			

**Table 3:** *N* = 1,000 for each country.

Country	Gender and Age profile by country.				
	Age 18–44	Age 45–64	Age 65–99	Female	Male
Sweden	346	354	300	507	493
Spain	373	355	272	488	512
Italy	351	352	297	497	503
Germany	352	354	294	496	504
France	351	351	298	489	511
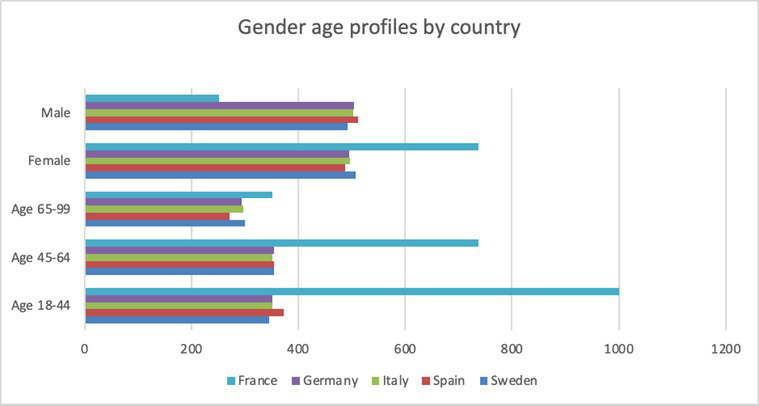					

**Table 4:** Income profile by country.

Country	0–30,000€	30,001–60,000€	60,001–120,000€	> 120,000€
Sweden	453	267	75	22
Spain	529	356	55	6
Italy	453	367	75	22
Germany	420	425	94	15
France	472	425	94	15
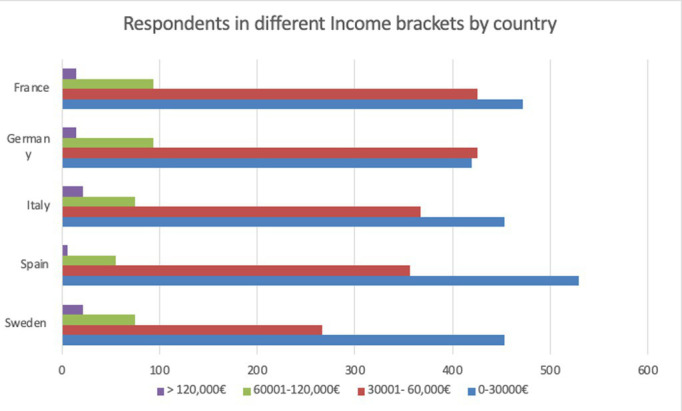				

**Table 5:** No of respondents by country living in different population areas.

Country	Less than 1,000	1,001–5,000	5,001–10,000	10,001–50,000	50,001–100,000	100,001–500,000	Over 500,000
Sweden	93	79	73	214	167	176	198
Spain	26	55	79	218	135	228	259
Italy	32	91	106	319	163	126	163
Germany	54	117	100	258	127	165	179
France	116	195	152	265	104	85	83
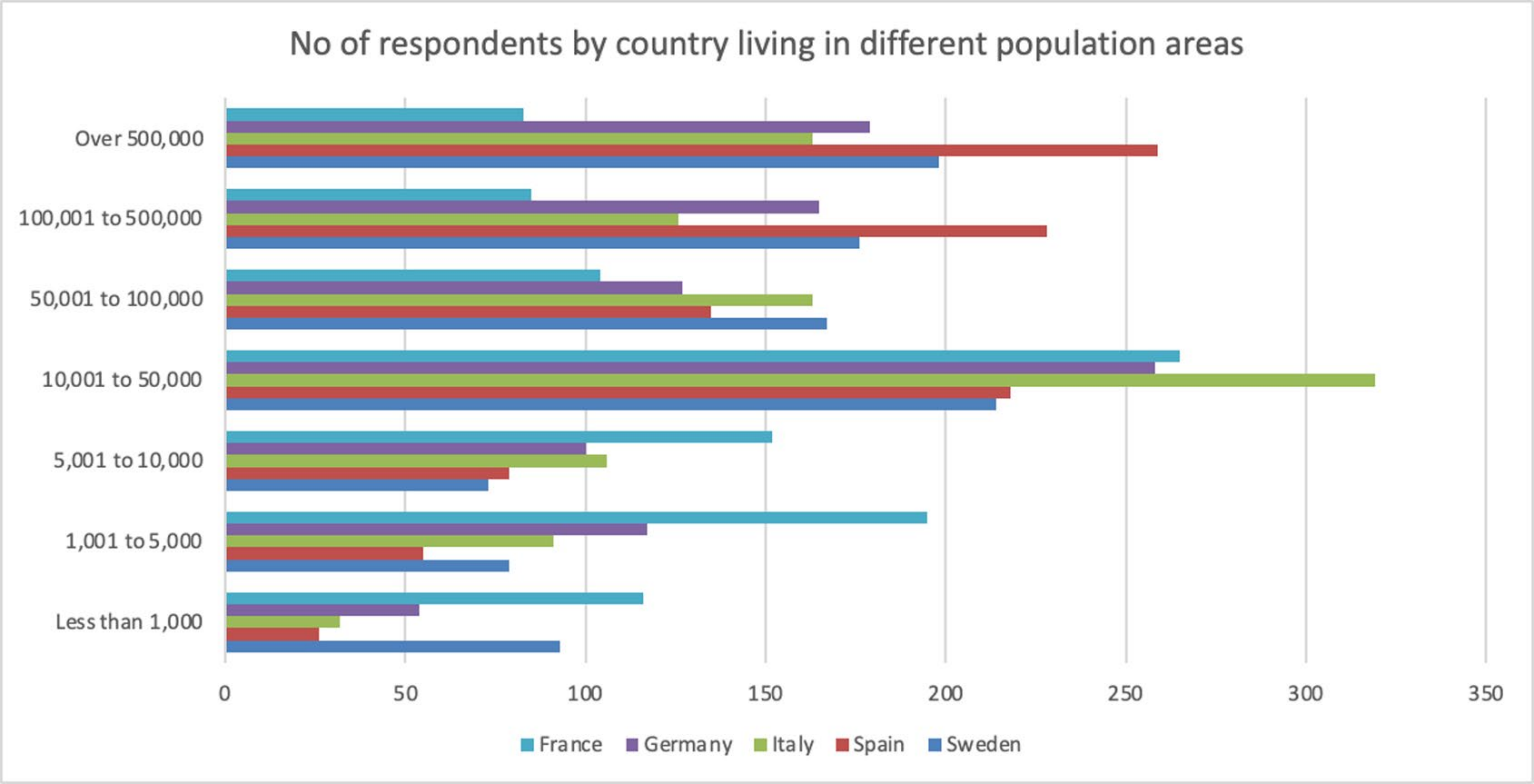							

### 3.1 Convenience

Sixty-nine percent (69%) of the survey respondents rated the opportunity to order prescription medicine online at any time as “beneficial” or “very beneficial.” This share is largest with 74% in Sweden and lowest with 59% in France. Furthermore, 67% of the respondents point out that direct delivery, a service inherent to ordering online, is perceived as “beneficial” or “very beneficial.” The highest approval ratings were found in Sweden (70%) and the lowest in France (58%).

Acknowledging the substantial time savings and increased flexibility provided by online access to prescription medicine, four groups of individuals indicate the highest perception for accessing online access to prescription medicine: chronically ill people, their caregivers, individuals with longer travel distances to physical pharmacies, and individuals who perceive the opening hours of pharmacies as inconvenient. These findings are revealed in columns (1) and (2) of Table 6. The two columns show estimated effects from an OLS analysis on the propensity to order prescription medicine online, our main outcome variable, which we interpret as demand for online access.

**Table 6:** Demand drivers.

Demand drivers	Dependent Variable				
	Propensity to obtain prescription medicine online (in sd)		Number of pharmacy visits	Duration per visit (in min)	Items bought
	(1)	(2)	(3)	(4)	(5)
Chronic condition	0.0915*** (0.0293)	0.126*** (0.0324)	7.67788 (0.591)	3.3905*** (0.863)	0.670*** (0.0424)
Caregiver	0.298*** (0.0307)	0.14.7*** (0.0334)	12.06*** (0.700)	8.913*** (0.929)	0.424*** (0.0467)
Long distance to pharmacy	0.0395** (0.0167)	0.0602*** (0.0178)			
Inconvenience from short opening hours	0.124*** (0.0173)	0.0644*** (0.0183)			
Constant	−0.499*** (0.0404)	−1.181*** (0.106)	3.070 (2.268)	17.53*** (2.680)	2.123*** (0.124)
Controls	No	Yes	Yes	Yes	Yes
Observations R2	4,673 0.051	3,577 0.218	3,577 0.244	3,577 0.106	3,577 0.177

The table shows the regression results of five different linear regression models: Models 1 and 2 show effects on the propensity to obtain prescription medicine online. While model 1 does not include control variables, model 2 does. Model 3 shows estimated linear effects on the number of pharmacy visits, model 4 on the duration of visits, and model 5 on the number of purchased items. For all three models, we include control variables. The control variables are country dummies, age, gender, income, education level, city size, and the frequency of general online shopping. We use robust standard errors. *, **, and *** mark significance levels of *p* < 0.05, *p* < 0.01, and *p* < 0.001, respectively.

The analyses in columns (1) and (2) demonstrate that the effect on the propensity to obtain prescription medicine online is statistically significant for both groups independent of model specification. Building on the preferred model specification with control variables, model (2), the effect is economically meaningful, with effect sizes of 0.13 sd (*p* < 0.001; 95%CI: 0.06–0.19) and 0.15 sd (*p* < 0.001; 95%CI: 0.08–0.21), respectively. Assuming that these detected effect sizes are accurate, the study was well-powered. A power analysis based on the effect sizes detected in regression model (2) reveals that there is more than 95% power to detect the effects related to chronically ill individuals and their caregivers. Even for the slightly smaller effects observed in the regression model (1), the power levels remain above 85%. Therefore, one can be confident that the significant results observed are not due to chance^22^.

Columns (3), (4) and (5) of Table 6 provide further insights into why these two groups indicate an increased desire for online access. The three columns present regression results from three different analyses, which are regressions with different dependent variables. The data reveal that patients with a chronic condition and their caregivers report an average of 7.7 (*p* < 0.001; 95%CI: 6.51–8.84) and 12.1 (*p* < 0.001; 95%CE: 10.68–13.43) additional pharmacy visits per year, respectively when compared to individuals without chronic conditions or caregiving duties. They report spending 3.4 min (*p* < 0.001; 95%CI: 1.70–5.09) and 8.9 min (*p* < 0.001; 95%CI: 7.09–10.73) longer per visit, respectively, and they purchase more items (0.67 and 0.42, respectively). As a result, chronically ill patients and their caregivers exhibit a notably increased preference for online access.

Coming back to the core analysis on demand, columns (1) and (2) of Table 6, further reveal that two other groups benefit from online access. It was found that respondents with longer travel distances to the next pharmacy and respondents who perceive the opening hours of physical pharmacies as inconvenient indicated a higher desirability for online access to prescription medicine. While the effects predicted by the model (2) are smaller in size with 0.06 sd (*p* = 0.001; 95%CI: 0.03–0.10) and 0.06 (*p* < 0.001; 95%CI: 0.03–0.10), respectively, they are still statistically significant, highlighting the fact that online access is perceived as a more convenient and flexible solution than visits to a physical pharmacy.

Lastly, the role of sociodemographic factors was explored — that is, those factors that were employed as control variables, see analysis in Appendix Table A1. It can be seen that gender, age, and income affect desirability for online access to prescription medicines. Women demonstrate a higher demand for online access to prescription medicine than men. This is highest for younger generations (represented by the age group 18–44) and decreases consistently for older generations. This finding can likely be attributed to varying degrees of technological proficiency or trust in online platforms across different age demographics. A higher propensity to obtain prescription medicine online among frequent online shoppers was seen. Through frequent purchasing, they may have developed enhanced trust in online services and delivery. Finally, it was observed that higher income levels are associated with an increased propensity to order prescription medicine online.

### 3.2 Health benefits

The second notable benefit of online access to prescription medicine, as indicated by the data, is a potential enhancement of health outcomes via improved medication adherence. Patients with a chronic condition require taking that medication regularly and on time, which necessitates regular pharmacy visits.

The data reveals that during the 6 months preceding our survey, 21% of the respondents with chronic illnesses encountered at least one incident of delayed medication intake. While several factors may lead to such non-adherence, a significant proportion of these incidents are attributed to time constraints faced by patients in refilling their prescriptions^23^. Specifically, 35% of the patients who reported adherence problems (equivalent to 7% of all respondents with chronic conditions) identified time constraints in refilling medication as an “important” or “very important” contributor to their non-adherence.

These issues of non-adherence might be alleviated through the online procurement of prescription medicine (e.g., (5)). Our analysis indicates that in Sweden and Germany, where patients already have online access to pharmacies, instances of non-adherence due to time constraints for medication refills are significantly lower. Specifically, the likelihood of this type of non-adherence is reduced by 22 percentage points (*p* < 0.001: 95%CI: 0.153–0.298).

Additionally, we observe that the respondents with chronic conditions who face challenges in timely medication refills themselves believe that their adherence would improve with the ability to order medicine online. Relative to their current likelihood of following a prescribed medication plan, they anticipate their adherence probability to increase by almost three percentage points (*p* = 0.02; 95%CI: 0.37–4.63) after ordering their medicine online. This is important as the result applies to all countries and not just those where online access is already available.

The former result pertains to an implicit test. Each respondent with a chronic condition was asked twice to indicate their likelihood of adhering to a prescribed medication plan: First, they were asked for their current likelihood of adhering to the medication plan. Then, they were asked for their estimated likelihood of adhering after gaining online access. As a result, it was possible to calculate the difference between both measures for those respondents who did not already order their medicine online. However, a more explicit response from our survey respondents located in Sweden and Germany was gained. Respondents were asked to indicate to what extent they believed that ordering online and receiving the medicine delivered to a preferred location would help in adhering to their prescribed medication plans. Among those who experienced non-adherence due to time constraints in refilling prescriptions, 62% believe that ordering online and 68% believe that delivery would help “much” or “very much.” For the belief about ordering online, this share is 30 percentage points (*p* < 0.001; 95%CI: 19.92–40.93) higher than for respondents with a chronic condition but no adherence problems. For the belief about the delivery effect, the share is 21 percentage points (*p* < 0.001; 95%CI: 9.90–32.74) higher.

Based on this line of evidence, it is not surprising that chronically ill individuals who report adherence problems due to time constraints refilling their prescriptions exhibit a greater desirability for online access to prescription medicine. 64% of these individuals report being “likely” or “very likely” to order prescription medicine online, which indicates an increase of 30 percentage points (*p* < 0.001; 95%CI: 23.5–36.4) relative to other respondents with a chronic condition. Moreover, using the full range of the 5-point Likert response scale, and controlling for our standard set of background variables, we find that the propensity to obtain prescription medicine online is 0.4 sd (*p* < 0.001; 95%CI: 0.258–0.531) greater for this subgroup. This effect is not only statistically but also economically significant (Table 7).

**Table 7:** Effects of non-adherence due to time constraints to refill medicine.

	Dependent Variable	
	Propensity to obtain prescription medicine online (in sd)	
	(1)	(2)
Non-adherence due to time constraints to refill medicine	0.506*** (0.0675)	0.4117** (0.0681)
Constant	−0.0114 (0.0191)	−0.860*** (0.130)
Controls	No	Yes
Observations	3,158	2,433
*R*2	0.022	0.239

The table shows the regression results of two linear regression models. Both models show the effect of non-adherence due to time constraints refilling prescriptions on the propensity to obtain prescription medicine online. Model 1 does not include control variables, model 2 does. Effects are measured in standard deviation. We use robust standard errors. *, **, and *** mark significance levels of *p* < 0.05, *p* < 0.01, and *p* < 0.001, respectively.

## 4 Barriers to broader adoption

The results presented in the previous section show there exists a significant interest for online access to prescription medicine across Europe and that this demand is mainly driven by certain vulnerable groups - chronically ill people and their caregivers. Furthermore, respondents with longer travel distances to the next pharmacy and respondents who perceive the opening hours of physical pharmacies as inconvenient also indicate a higher demand for online access to prescription medicine.

Despite the increasing interest in online access to prescription medicines, several barriers continue to limit patient access and utilization. In the European Union, existing regulations offer necessary safeguards, but two primary obstacles are likely to impact adoption: limited awareness and constraints on the digital growth of pharmacies (15).

### 4.1 Lack of awareness

All findings on the demand for online access to prescription medicine presented in Section 3 were based on what participants knew about online pharmacies before our study. The initial knowledge about online pharmacies, however, was poor. Indeed, before participating in the survey: 77% of the respondents were not aware of the EU Common Logo; 53% were unaware that authorized pharmacies selling online are subject to the same safety/security protocols as their counterparts that only sell at a brick-and-mortar store.

Upon informing the participants about the Common Logo and the regulations governing online pharmacies, a notable shift in their propensity towards obtaining prescription medicine online was observed in France, Germany, Italy, and Spain. Our information intervention led to an average increase in the propensity of respondents to procure prescription medications online by 0.33 sd (*p* < 0.001; 95%CI: 0.30–0.35)^24^. Although this evidence does not establish a causal relationship, it suggests that increased awareness may reduce initial skepticism and enhance the adoption of online services for prescription pharmacy products.

Reflecting on awareness and its impact on the use of online access, it is also important to consider how limited awareness correlates with individuals’ concerns in regions where online access is not yet available. In France, Italy, and Spain, the survey results reveal a significantly higher level of concern regarding issues such as medicines not being delivered (with 65% of respondents in these countries expressing worry, compared to 36% in Germany and Sweden; *p* < o.001) or arriving late (67% vs. 43%; *p* < 0.001). Additionally, there exists a greater fear of receiving counterfeit medicines (62% vs. 27%; *p* < 0.001). These concerns are notably more pronounced than in Germany and Sweden. Although some of these concerns might reflect a broader distrust in institutions within France, Spain, and Italy, the significant discrepancies could also imply that a lack of knowledge about the operations of online pharmacies contributes to apprehensions.

### 4.2 Constraints on the digital expansion of pharmacy operations

Providing online pharmacy services may create challenges for small-scale pharmacies. A small scale pilot survey was conducted among pharmacy owners to understand what constraints they face when serving patients online. To so do, we systematically gathered the views of 25 pharmacy owners across the same set of Member States. We sought a sample that could capture the views both of physical pharmacy owners without any intention to operate online, in addition to some that have started some online operations^25^. Thus, among 25 respondents there are 13 pharmacies with and 12 without online operations. In these interviews, it emerged that, on average, a pharmacy owner has 3.6 employees and operates in one location, or, in some cases, two^26^. This aligns with the fact that pharmacies in the EU operate predominantly as small and medium-sized enterprises (SMEs).

Half of the sample were pharmacy owners who do not yet engage in online sales; they indicated a lack of the necessary technology and marketing skills as one of the key challenges. These barriers are not shared by pharmacy owners who already provide online pharmacy services. Moreover, pharmacy owners identified the absence of a simple solution for delivery logistics as another key challenge. However, unlike technology and marketing skills, this challenge is shared among all pharmacy owners, regardless of whether they already operate online or not. At the same time, these are well-documented barriers that small businesses face when venturing online (17, 20). In conclusion, our pilot survey (with inherent limitation of a sample size of 25 pharmacists being interviewed) clearly shed light on the current barriers as described by the pharmacists.

## 5 Discussion

The survey data reveals (i) indicate a preference for more flexibility when purchasing prescription medicine and highlight the: increased convenience of obtaining a medicine online (ii) a significant interest in ordering prescription medicine online, (iii) that online access to prescription medicine entails a substantial, untapped potential in terms of convenience benefits, (iv) that these benefits include potential health improvements and that (v) the desirability for online access to prescription medicine is likely to increase over time, as knowledge about the regulations of online pharmacies increases and initial unwarranted concerns fade. Consequently, these insights support an expansion of online access to allow Europeans in more countries to order prescription medicine online. However it is noted that the respondents were individuals who have procured goods and services through online channels and who obtained prescription medicine in the past year and on chronic patients. And so the survey did not cover those that had not carried out such activity.

The study also revealed that online access allows for more control over the purchase of prescription medicine. It enables individuals to better integrate purchasing duties into their daily routine by ordering at any time they want. Second, it reduces the necessary effort to collect medicine and allows for considerable time savings. Comparing the estimated time for an online purchase to that of a visit to a physical pharmacy in countries where online access is permitted, we find that individuals save about 15 min^27^. Since the average person visit a pharmacy almost 16 times per year, this time saving is substantial as it amounts to annual savings of 240 min (i.e., 4 h).

Equally, the evidence on barriers, emerged as part of the above evidence gathering, confirms the value of accompanying and safeguarding consumers as they use their existing online skills (or newly gained skills) into a new domain for them, namely accessing prescription medicines online (if they live in countries where this has been blocked so far). The same can apply to barriers that pharmacy businesses face as they consider and execute digital transformation, which is a standard challenge already experienced (and considerably overcome) when traditional (offline) consumer-facing organizations have started embracing digital channels in their way of, e.g., serving consumers/patients.

Online access to prescription medicines offers immediate convenience for chronically ill individuals and their caregivers. Calculations indicate that time savings alone could create economic value between €1.3 billion and €2.3 billion annually in the EU if all 19 member states currently prohibiting online access were to allow it^28^. Beyond convenience, the health benefits from improved adherence also present substantial value, as societal costs due to non-adherence are currently estimated at €125 billion per year in Europe (12). Future studies could further explore the practical implications of online access on adherence.

For these benefits to materialize, removing restrictions to online access should further be complemented by information campaigns to mitigate initial unwarranted concerns and enhance knowledge of existing safeguards applicable to the online dispensing of prescription medicines by registered pharmacies. Notably, concerns about non-delivery, delayed delivery, or counterfeit medicines are higher in countries where online access is currently prohibited (e.g., France, Italy, and Spain) compared to those where it is allowed (e.g., Germany and Sweden). We interpret this result as indicating that people’s concerns are considerably mitigated once they use themselves or experience their friends and family using legitimate online pharmacy services. This study also revealed that once a respondent knew that the supply chain for an prescription medicine obtained online was dispensed under the same conditions as a physical pharmacy environment, then this augmented the likelihood of a patient adopting an online option. In parallel it can be conceivable that broader access to regulated suppliers of prescription medicines can discourage European consumers from falling into the harms of unlicensed suppliers that attempt to distribute online against the law. Therefore, further research could test the above crowding-out effect where licensed, regulated supplied channels and products chase out their illegal counterparts on the internet.

The results can be generalized to countries with a robust regulatory framework for online access that allow consumers to identify legitimate online retailers and addresses the risks of selling counterfeited products, as is the case in the EU today.

We acknowledge that the insights presented in this article are based on correlational evidence rather than causal inference. The results suggest that chronically ill individuals who report non-adherence due to time constraints in refilling their prescriptions are more likely to use online access to obtain medication. This indicates that providing online access could help address non-adherence caused by such time constraints. The analysis further reveals that in Sweden and Germany, where patients already have online access to pharmacies, instances of non-adherence due to time constraints for medication refills are significantly lower compared to countries where online access to prescription medicines is prohibited. Adherence may also be influenced by several factors related to the patient, treatment, or healthcare provider (see, e.g., Agh). While this evidence suggests a link between adherence and online access to prescription medication, further research would be needed to ascertain causal and other correlational links between online access and adherence.

Furthermore, the sample was not designed to screen or achieve balanced representation of rural vs. urban residence (for budgetary reasons we had to prioritize a sample being representative to even more key demographic dimensions). The potential benefits in terms of convenience for individuals living in rural areas may be significant because they have longer distance to travel to the closest pharmacy when compared to those living in cities and towns. Future research on online access to prescription medicines could usefully spend renewed effort on individuals living in rural areas, where geographical isolation and limited healthcare infrastructure often restrict access to necessary medications. In these communities, patients may face challenges such as long travel distances to the nearest pharmacy, limited transportation options, and fewer healthcare providers, all of which can hinder timely access to prescription drugs. Exploring the effectiveness and potential barriers to online prescription services in rural settings could illuminate critical insights into how digital healthcare innovations can bridge these gaps.

## 6 Conclusion

This study underscores the advantages of online access to prescription medicines, particularly for chronically ill patients and their caregivers. The research demonstrates that such access can improve medication adherence, reduce the incidence of non-adherence due to time constraints, and offer substantial convenience through time savings and flexibility. However, restrictions on online access, lack of awareness and constraints on the digital expansion of pharmacies continue to impede broader adoption in many EU Member States.

To harness these benefits, it is important to implement regulatory reforms that facilitate online access to prescription medicines and targeted information campaigns. Such measures can improve patient convenience and have the potential to enhance health outcomes reducing overall healthcare costs across Europe.

Given the evidence presented in this paper, policymakers should seriously consider taking the necessary steps to enable online access. This could be achieved by amending Article 85(c) of the Community Code of medicinal products for human use^29^, to encourage Member States to permit online access to prescription medicines through authorized pharmacies, in line with the laws and regulations of the Single Market. Expanding online access to medicines complements the services of physical pharmacies, supporting an omnichannel approach that enhances convenience and flexibility for consumers.

Furthermore, the research shows that having legitime online access options to prescription medicines can increase trust among EU citizens and help them identify and use authorized online pharmacies and retailers. Allowing access through these authorized online channels could therefore prevent patients from accidentally accessing unauthorized sites, thus avoiding the risk of obtaining falsified medicines.

Given that the EU has long-established common systems to safeguard the purchases of medicines online, policymakers and industry should consider strengthening consumer awareness of existing safeguards. For Member States, this involves fulfilling obligations under Article 85d of the Falsified Medicine Directive^30^. While some Member States have partially fulfilled the spirit of this article^31^, recent analysis tends to question these efforts^32^. Educating the public about the need to obtain medicines from an authentic online source is paramount in the fight against falsified medicines. Removing misunderstandings and clarifying questions — which are still commonplace amongst consumers as we have found — through awareness campaigns will allow patients to make an informed choice when using online channels to access prescription medicines.

## Data Availability

The datasets presented in this article are not readily available because requests for access will be considered on a case-by-case basis, depending on the intended use and the nature of the requesting organization. Requests to access the datasets should be directed to mike.isles@asop.eu.

## Footnotes

^1^With e-prescriptions, error rates in dispensing medications could decrease by an average of 6%, rising to 15% in digitally advanced countries. See European Commission, A European Health Data Space: harnessing the power of health data for people, patients and innovation (Communication) COM (2022) 196 final.

^2^Survey on Citizens’ appreciation of the option to access prescription medicines Online https://www.eaep.com/uploads/survey-on-citizens-appreciation-rx-online.pdf?_cchid=d152fe42751f6b5afc59b5f354229492.

^3^ https://www.eaep.com/uploads/executive-summary-iegus-report-on-the-role-of-online-pharmacies-on-the-supply-of-pharmaceuticals-in-germany.pdf?_cchid=1ebaf82a6bc8e1da2a60d97a963c3c2e

^4^NHS App. Available online at: https://www.nhs.uk/nhs-app/.

^5^Illicit Trade in Counterfeit Pharmaceutical Product report.1–93.Available online at: https://buysaferx.pharmacy//wp-content/uploads/2020/03/Trade_in_Counterfeit_Pharmaceutical_Products_en.pdf.

^6^WHO, A Study on the Public Health and Socioeconomic Impact of Substandard and Falsified Medical Products (Geneva, 2017); WHO, WHO Global Surveillance and Monitoring System for Substandard and Falsified Medical Products (Geneva, 2017); and The Globalization of Crime: A Transnational Organized Crime Threat Assessment (United Nations publication, Sales No. E.10.IV.6).

^7^World Health Organization (WHO), Report by the Director-General on the Member State mechanism on substandard/spurious/ falsely-labelled/falsified/counterfeit medical products, Report of the Director-General, document A70/23, annex, appendix 3.

^8^Combatting falsified medicine related crime UNODC https://buysaferx.pharmacy//wp-content/uploads/2019/05/Guide_Falsified_Medical_Products.pdf.

^9^Rogue Rx Activity report https://nabp.pharmacy/wp-content/uploads/2020/05/Rogue-Rx-Activity-Report-May-2020.pdf.

^10^2021 Survey American perceptions and use of online pharmacies https://buysaferx.pharmacy//wp-content/uploads/2021/07/ASOP-Global-Foundation-2021-Consumer-Behavior-Survey-Key-Findings_Final-7.9.2021.pdf.

^11^An educational Google Adword campaign ran in five European countries where an online survey recorded 2,300 respondents and 91% having been informed of the plethora of websites selling medicines illegally said they would change their behavior and seek out a legitimate pharmacy. https://eaasm.eu/wp-content/uploads/EAASM-ASOP-EU-educational-website-map.pdf.

^12^See Fittler et al. (13) for an overview of potential challenges to online sales of medicines.

^13^The Falsified Medicines Directive (Directive 2011/62/EU) enacted in 2011 puts forward principles and rules to limit the spread of falsified medicines.

^14^In 2014, the EU introduced into the Falsified Medicines Directive an obligatory EU Common Logo linked to the national register of authorized online pharmacies and retailers, enabling consumers to identify authorized sources of medicines.

^15^The Community Code of Medicines of 2001 (Directive 2001/83/EC) relating to medicinal products for human use, sets up minimum criteria and standards for online pharmacy dispensing, covering both over the counter and prescription medicines.

^16^European Commission, Commission welcomes final agreement of EU Digital Identify Wallet, Press Release, 8 November 2023, https://ec.europa.eu/commission/presscorner/detail/en/ip_23_5651.

^17^All respondents are at least 18 years old.

^18^35% of the EU population suffers from a chronic condition (Eurostat, online table: hlth_silc_11). Our sample includes a 63% share of this category.

^19^In 2023, 92% of the surveyed individuals used internet and 70% (Eurostat, online table: isoc_ci_ifp_iu) bought or ordered products or services online in the 12 months preceding the survey (Eurostat, online table: isoc_ec_ibuy).

^20^Source: Eurostat (online data code: isoc_ci_ifp_iu).

^21^Eurostat (online data code: isoc_ec_ibuy and isoc_ec_ib20).

^22^We note that the power of our analysis exceeds the standard level of 80%. This high power is attributed to our large sample size, which enables the detection of even smaller effects. Since our survey did not involve ethical concerns and was not limited by financial constraints, we were able to recruit a larger sample. We consider this a strength of our study.

^23^The three most important contributors as indicated in our survey are (i) medicine not in stock of pharmacy, (ii) forgetting to refill the medication and (iii) time constraints to refill the medication.

^24^In Sweden, where online access to prescription medicine is already used frequently and knowledge about the regulation of online pharmacies is more widespread (with 63% of respondents indicating awareness that authorized online pharmacies operate under the same regulations as physical pharmacies), no significant change was observed.

^25^Like in the case of the consumer survey, the structured interviews were conducted by a market research company. We used computer-assisted telephone interviewing technique. Pharmacies were selected randomly from the business register, for physical pharmacies, or from the European list of online medicine retailers: available at the European Medicine Agency website, see link.

^26^Out of 25 pharmacy owners interviewed only two report more than 2 locations.

^27^This result is calculated as the difference between the estimated time of the last pharmacy visit (indicated by all respondents in our sample) and the reported time of an online purchase (indicated by respondents in Sweden and Germany who have previously ordered prescription medicine online).

^28^For the calculation, please see Jervelund et al. (21). Unlocking the benefits of online access to prescription medicines across the EU. Copenhagen Economics.

^29^Directive 2011/62/EU of the European Parliament and of the Council of 8 June 2011 amending Directive 2001/83/EC on the Community code relating to medicinal products for human use, as regards the prevention of the entry into the legal supply chain of falsified medicinal products Text with EEA relevance.

^30^This article mandates that the European Commission, in collaboration with the European Medicines Agency and national authorities, conduct public information campaigns highlighting the risks of falsified medicines and educating consumers on the use of the common logo, Member State websites, and the Agency’s website to identify authentic online sources.

^31^Fighting Fakes by Member States Alliance for Safe Online Pharmacy January 2018 Falsified Medicines and the Common Logo – raising public awareness, see link.

^32^Report on EU / European Countries’ FMD Common Logo Survey, see link.
