# Supplementary material for: The benefits of online access to prescription medicines: the European patient’s perspective
Source: Front Public Health. 2025 Sep 8;13:1513338. doi: 10.3389/fpubh.2025.1513338 (PMC12451712; doi:10.3389/fpubh.2025.1513338)
Supplement: Supplementary file 1 [file Data_Sheet_1.pdf]

Survey

Introduction

Dear Participant,

In this survey, we would like to hear about your experience with getting your prescription medicine. We aim to learn how you currently manage your prescription needs and identify any unmet needs in this regard. Finally, we will seek your views on whether you would be comfortable and able to buy online your prescription medicine if this is [DE, ES] | would become possible.

Any information you provide us with will be treated as confidential, it will be combined with survey replies from others like yourself.

We sincerely thank you for taking the time to complete this survey.

--------------------- --------------------------- page break --------------------------- ------------------------

General questions

1. When was the last time you obtained prescription medicine (independent of whether that medicine was for yourself or a person you care for)?
   (Label: Obtain last time)
   - Within the last week
   - Within the last month (but more than a week ago)
   - Within the last 3 months (but more than a month ago)
   - Within the last 6 months (but more than 3 months ago)
   - Within the last year (but more than 6 months ago)
   - More than a year ago

2. Do you have a chronic condition that requires a repeat prescription of medicine or medicines? A chronic condition is defined as a condition that lasts 1 year or more and requires ongoing medical attention (e.g., asthma or diabetes).
   (Label: Chronic Condition Self)
   - Yes -> ChronicSelf == 1
   - No -> ChronicSelf == 0
   - Don't know -> ChronicSelf == 0
   - Prefer not to answer -> ChronicSelf == 0

3. Do you obtain prescription medicine for someone you care for?
   (Label: Obtain medicine)
   - Yes -> ObtainedOther == 1
   - No
   - Prefer not to answer

4. [If ObtainedOther == 1] Does the person you care for have a chronic condition that requires a repeat prescription of medicine(s)?
   (Label: Chronic Condition Other)
   - Yes -> ChronicOther == 1
   - No -> ChronicOther == 0
   - Don't know -> ChronicOther == 0
   - Prefer not to answer -> ChronicOther == 0

--------------------- --------------------------- page break --------------------------- ----------------------

5. [If ObtainedOther == 1] Please select the option that best describes the relationship to the person you care for. Please select all that apply.
(Label: Caregiver)
- I care for a dependent child.
- I care for an elderly person in my family.
- I care for a friend.
- Caregiving is my job.
- I care for someone else. (Please specify)

--------------------- --------------------------- page break --------------------------- -----------------------

Current dispensing of prescription medicine

Physical pharmacies

6. How convenient is it for you to obtain a prescription medicine from a physical pharmacy?
(Label: Convenience of Pharmacy Visit)
- Very convenient
- Convenient
- Neither convenient nor inconvenient
- Inconvenient
- Very inconvenient

--------------------- --------------------------- page break --------------------------- -----------------------

7. What makes it convenient or inconvenient for you to obtain a medicine from a physical pharmacy? Please rate the following items from 1 (very inconvenient) to 5 (very convenient).
(Label: Factors of Pharmacy Visit)

	1	2	3	4	5
Distance to next physical pharmacy	◎	◎	◎	◎	◎
Opening hours of nearby physical pharmacies	◎	◎	◎	◎	◎
Pharmacist interaction	◎	◎	◎	◎	◎
Revealing health conditions to third persons	◎	◎	◎	◎	◎
Confidentiality of pharmacy staff	◎	◎	◎	◎	◎
My physical condition	◎	◎	◎	◎	◎

--------------------- --------------------------- page break --------------------------- -----------------------

8. Thinking of the last time you obtained a prescription medicine at a pharmacy (independent of whether that medicine was for yourself or a person you care for), how much time did that take you? Please count the time for travel and the time you spent waiting and receiving service in the pharmacy.
(Label: Time Pharmacy Visits)

-- [sliding bar with an interval of 5 minutes max 120]

9. Thinking of the last time you ordered any prescription medicine at a pharmacy, how many products did you buy in total?
(Label: Number of items bought)

- 1
- 2
- 3
- 4
- More than 4

---------------------- ---------------------------- page break ---------------------------- ------------------------

10. How long does it take you to travel to the nearest pharmacy from where you live?
(Label: Time to the Nearest Pharmacy)

-- [sliding bar with an interval of 5 minutes]

---------------------- ---------------------------- page break ---------------------------- ------------------------

11. How often have you had to visit a pharmacy during the last 12 months to obtain a prescription medicine (independent of whether that medicine is for yourself or a person you care for)?
(Label: Frequency Pharmacy Visits)

- More than once per week
- Once per week
- Once every two weeks
- Once per month
- Once every two months
- Once every three months
- Once per year
- Never
- I usually obtain my prescription medicine online [DE, SE].

---------------------- ---------------------------- page break ---------------------------- ------------------------

12. When you obtain a prescription medicine at the pharmacy, how often do you also buy over-the-counter medicine at the same time? Over-the-counter medicine is a medicine that does not require a prescription by a medical doctor (for example, simple painkillers or cough and cold remedies).
(Label: Combine Rx and OTC)

- Always (10 out of 10 times)
- Often (7-9 out of 10 times)
- Sometimes (4-6 out of 10 times)
- Rarely (1-3 out of 10 times)
- Never (0 out of 10 times)

---------------------- ---------------------------- page break ---------------------------- ------------------------

13. How frequently do you seek advice from a pharmacist regarding …
(Label: Pharmacist Advice)

	Very often	Often	Sometimes	Rarely	Never
Obtaining a prescription medicine	◎	◎	◎	◎	◎

Purchasing over-the-counter medicine (e.g., painkillers) ◎ ◎ ◎ ◎ ◎

---------------------- ---------------------------- page break ---------------------------- ------------------------

14. How often does another person go to the pharmacy for you to obtain a prescription medicine?
(Label: Caregiver Support)
  - Always (10 out of 10 times)
  - Often (7-9 out of 10 times)
  - Sometimes (4-6 out of 10 times)
  - Rarely (1-3 out of 10 times)
  - Never (0 out of 10 times)

-- page break ---------------------------- ------------------------

Online pharmacies

15. Have you ever bought over-the-counter medicine (e.g., painkillers) online (independent of whether that medicine was for yourself or another person)?
(Label: Experience with Online Medicine - General)
  - Yes
  - No
  - Don't know

16. [Ask If SE or DE. In IT, FR, and ES all will be OnlineBuyer==0] Have you ever ordered any prescription medicine online (independent of whether that medicine was for yourself or another person)?
(Label: Experience with Online Medicine - Prescription)
  - Yes -> OnlineBuyer == 1
  - No -> OnlineBuyer == 0
  - Don't know -> OnlineBuyer == 0
  - Prefer not to answer -> OnlineBuyer == 0

-- page break ---------------------------- ------------------------

17. [if OnlineBuyer == 1] Thinking of the last time you ordered any prescription medicine online, how much time did it take you?
(Label: Time of Ordering Online)

-- [sliding bar with an interval of 1 minute with a max of 60min]

18. [if OnlineBuyer == 1] Thinking of the last time you ordered any prescription medicine online, how many products did you buy in total?
(Label: Number of items bought online)
  - 1
  - 2
  - 3
  - 4
  - More than 4

19. [if OnlineBuyer == 1] Thinking of the last time you ordered any prescription medicine online, how much time did it take to deliver?
(Label: Time of Delivery)

- Half a day
- One day
- Two days
- Three days
- Four days
- More than four days

--------------------- --------------------------- page break --------------------------- -----------------------

Disease management

Acute condition

20. Last time you needed a prescription medicine to treat a sudden illness or condition, how important was it for you to obtain it as quickly as possible?
    (Label: Delivery Speed)
    - 1 – not at all important
    - 2
    - 3
    - 4
    - 5 – Very important

--------------------- --------------------------- page break --------------------------- -----------------------

Chronic condition

21.

If ChronicSelf == 1

A. Given your chronic condition, how important is it for you to take your medicine consistently and on time?
(Label: Importance of Medicine)
- 1 – Not at all important
- 2
- 3
- 4
- 5 – Very important

If ChronicSelf == 0 AND ChronicOther == 1
B. Given the chronic condition of the person you are caring for, how important is it for them to take their medicine consistently and on time?
(Label: Importance of Medicine)
- 1 – Not at all important
- 2
- 3
- 4
- 5 – Very important

22.

If ChronicSelf == 1

A. How often do you need to refill your prescription medicine?
(Label: Refill Frequency)
- Once per week
- Once every two weeks
- Once per month
- Once every two months
- Once per quarter (every three months)
- Once per year

If ChronicSelf == 0 AND ChronicOther == 1
B. How often does the person you are caring for need to refill their prescription medicine?
(Label: Refill Frequency)
- Once per week
- Once every two weeks
- Once per month
- Once every two months
- Once per quarter (every three months)
- Once per year

--------------------- --------------------------- page break --------------------------- -----------------------

23. [If ChronicSelf == 1] In the past 6 months, have you encountered a situation where you were unable to take your medicine in time?

(Label: Adherence Past 6 Months I)

- Yes --> [Adherence in Past 6 Months == 1]
- No --> [Adherence in Past 6 Months == 0]
- Don’t know --> [Adherence in Past 6 Months == 0]
- Prefer not to answer --> [Adherence in Past 6 Months == 0]

--------------------- --------------------------- page break --------------------------- -----------------------

24. [if Adherence in Past 6 Months == 1] Why were you unable to take your medicine ? Please rate the importance of the following factors in contributing to you not taking your medicine. Rate each factor from 1 to 5, where 1 indicates 'Not at all important' and 5 indicates 'Very important.
(Label: Adherence Past 6 Months II)

	1	2	3	4	5
I forgot to refill my medicine	◎	◎	◎	◎	◎
My pharmacy did not have my medicine in stock	◎	◎	◎	◎	◎
I did not have the time to buy/refill the medicine	◎	◎	◎	◎	◎
My physical condition did not allow me to get to the pharmacy	◎	◎	◎	◎	◎
I did not see a need to take it	◎	◎	◎	◎	◎
The closest pharmacy was too far away	◎	◎	◎	◎	◎

--------------------- --------------------------- page break --------------------------- -----------------------

25. [If ChronicSelf == 1] How closely did you follow your prescribed medicine plan (i.e., take your medicine consistently and on time) in the last 6 months? Please use the slider below to respond on a scale from 0% to 100% where 0% means “did not follow at all” and 100% means “followed exactly”.
(Label: Adherence last 6 Months (III)

[add slider]

26. [If ChronicSelf == 1] Which factors help you to follow your prescribed medicine plan? Please rate the following factors from 1 to 5, where 1 indicates ‘not at all’ and 5 ‘very much’.
(Label: Adherence factors)

	1	2	3	4	5
Information about medicine	◎	◎	◎	◎	◎
Proximity to the pharmacy	◎	◎	◎	◎	◎
Possibility to discuss potential side effects with the pharmacist	◎	◎	◎	◎	◎
Close attention by my doctor to my condition					
Reminders to take the medicine	◎	◎	◎	◎	◎
Regular follow-ups to refill and re-order on time	◎	◎	◎	◎	◎
Having a stock of medicines at home	◎	◎	◎	◎	◎
Ordering my medicine online [SE, DE]	◎	◎	◎	◎	◎
Delivery to your preferred location [SE, DE]	◎	◎	◎	◎	◎

---------------------- ---------------------------- page break ---------------------------- ------------------------

Online sales of prescription medicines

Demand

27. [DE SE == 0] How likely would you be to order a prescription medicine online if given the opportunity?
(Label: Likelihood of Online Purchase - General)
    - 1 – Very unlikely
    - 2
    - 3
    - 4
    - 5 – Very likely

    [DE SE == 1] How likely are you to order a prescription medicine online?
    (Label: Likelihood of Online Purchase - General)
    - 1 – Very unlikely
    - 2
    - 3
    - 4
    - 5 – Very likely

---------------------- ---------------------------- page break ---------------------------- ------------------------

Benefits

28. [OnlineBuyer == 0] Imagine the pharmacy you normally visit starts to sell your prescription medicine online and to deliver it to your preferred location (home address or pick-up point). How would you rate the following features of such a service on a scale from 1 to 5, where 1 indicates "Not at all beneficial" and 5 indicates "very beneficial".
(Label: Value Factors)

	1	2	3	4	5
Ordering at any time	◎	◎	◎	◎	◎
Time saved on visiting pharmacies	◎	◎	◎	◎	◎
Remaining anonymous	◎	◎	◎	◎	◎
Real-time medicine availability	◎	◎	◎	◎	◎
Ease of seeing prices	◎	◎	◎	◎	◎
Direct delivery to your preferred location (home or pick-up point)	◎	◎	◎	◎	◎
Automated refills that ensure consistent medicine supply at your home	◎	◎	◎	◎	◎

[OnlineBuyer == 1]. How do you rate the following features of ordering online a prescription medication on a scale from 1 to 5, where 1 indicates "Not at all beneficial" and 5 indicates "very beneficial".
(Label: Value Factors)

	1	2	3	4	5
Ordering at any time	◎	◎	◎	◎	◎
Time saved on visiting pharmacies	◎	◎	◎	◎	◎
Remaining anonymous	◎	◎	◎	◎	◎
Real-time medicine availability	◎	◎	◎	◎	◎

Ease of seeing prices	◎	◎	◎	◎	◎
Direct delivery to your preferred location (home or pick-up point)	◎	◎	◎	◎	◎
Automated refills that ensure consistent medicine supply at your home	◎	◎	◎	◎	◎

29. [If ChronicSelf == 1] [OnlineBuyer == 0] If you were to order your medicine online, how closely would you follow your prescribed medicine plan (i.e., take your medicine consistently and on time)? Please use the slider below to respond on a scale from 0% to 100% where 0% means "would not follow at all" and 100% means "would follow exactly".
(Label: Adherence with Online)

[add slider]

30. [OnlineBuyer == 0] Which of the following experiences from a visit at a physical pharmacy would you miss, if you were to order your medicine online? Please select all that apply.
    - Personal interaction with a pharmacist.
    - Expert advice from a pharmacist.
    - Immediate responses to potential questions.
    - Learning about new pharmacy products.
    - Physical receipts and documentation.
    - Opportunity to verify that I buy the correct medicine and the desired brand.
    - Opportunity to use the medicine immediately after I buy it.
    - Support of the local businesses.
    - None of the above.

--------------------- --------------------------- page break --------------------------- -----------------------

Perception of safety

31. [OnlineBuyer == 0] If you were to obtain a prescription medicine online, how concerned would you be about the following factors? Please rate them on a scale from 1 to 5, where 1 indicates "Not at all concerned" and 5 indicates "Very concerned".
(Label: Risk Factors)

	1	2	3	4	5
Receiving a fake medicine	◎	◎	◎	◎	◎
Medicine does not arrive	◎	◎	◎	◎	◎
Medicine does not arrive on time	◎	◎	◎	◎	◎
Wrong medicine or wrong dosage is delivered	◎	◎	◎	◎	◎
My personal data would be used for unauthorized purposes	◎	◎	◎	◎	◎

[OnlineBuyer == 1] Last time you ordered a prescription medicine online, how concerned were you about the following factors? Please rate them on a scale from 1 to 5, where 1 indicates "Not at all concerned" and 5 indicates "Very concerned".
(Label: Risk Factors)

	1	2	3	4	5
Receiving a fake medicine	◎	◎	◎	◎	◎

Medicine does not arrive	◎	◎	◎	◎	◎
Medicine does not arrive on time	◎	◎	◎	◎	◎
Wrong medicine or wrong dosage is delivered	◎	◎	◎	◎	◎
My personal data would be used for unauthorized purposes	◎	◎	◎	◎	◎

--- page break --------------------------- -----------------------

Marketplaces

32. If you could select a known or other 'local' pharmacy from an e-commerce shop, would you use this e-commerce shop to access the pharmacies?
    (Label: Use Marketplace)
    - Yes --> [Use Marketplace = 1]
    - No --> [Use Marketplace = 0]
    - Don't know --> [Use Marketplace = 0]

33. [if Use Marketplace = 1] Why would you use an e-commerce shop to access medicine from pharmacies? Please select all that apply.
    (Label: Use Marketplace - Y)
    - I am familiar with the websites of e-commerce shop.
    - e-commerce shop ensure that deliveries arrive on time.
    - I can combine my order with other orders from the same e-commerce shop.
    - I trust the prices and services of established e-commerce shop.
    - Other

    [if Use Marketplace = 0] Why would you not use an e-commerce shop to access medicine from pharmacies? Please select all that apply.
    (Label: Use Marketplace - N)
    - I am worried that my health data would be used for unauthorized purposes.
    - I fear that I could receive unsafe or fake medicine.
    - I do not think that the business model of an e-commerce shop is suitable for obtaining prescription medicines.
    - I do not want to support the business model of e-commerce shop in general.
    - Other

--- page break --------------------------- -----------------------

Awareness

34. Were you aware that medicines obtained via certified pharmacies operating online use the same safe and secure supply routes as physical pharmacies?
    (Label: Awareness of Regulations)
    - Yes
    - No

35. Did you know that legitimate online pharmacies have the following logo on their website [-> show logo]
    (Label: Awareness of Logo)
    - Yes
    - No

-------------------- --------------------------- page break --------------------------- -----------------------

36. Now that you are fully aware of the rules under which online pharmacies operate and that there exists the logo that allows you to identify a legitimate online pharmacy for ordering prescription medicine online, how likely would you be to order your medicine online?
    (Label: Change in Perception)
    - 1 – very unlikely
    - 2
    - 3
    - 4
    - 5 – very likely

-------------------- --------------------------- page break --------------------------- -----------------------

37. [If OnlineBuyer == 0] Imagine that the pharmacy that you normally visit starts to make available for you the same medicines with delivery to your preferred location (home or pick-up point). Imagine that this includes prescription medicines in a safe and secure way, certified and consistent with national regulations.
    How likely would you be to order your medicine online from this known local pharmacy?
    (Label: Safe & Secure, likelihood to order online)
    - 1 – very unlikely
    - 2
    - 3
    - 4
    - 5 – very likely

-------------------- --------------------------- page break --------------------------- -----------------------

Socio-demographics

38. How old are you in years? [IPSOS]
    (Label: Age)

39. What is your gender? [IPSOS]
    (Label: Gender)

40. Where do you live? [IPSOS]
    (Label: Region)

41. What is your total annual household income, before taxes?
    (Label: Income)

42. What is your highest level of education completed?
    (Label: Education)

43. What is your profession?
    (Label: Profession)

44. Approximately how many people live in the city/town/village you reside in?
    (Label: City Size)

45. How often do you make purchases online for any product of any time (e.g., clothing, electronics, groceries, etc.)?
    (Label: Online Shopping)
    - Multiple times per week

- Once per week
- Once every two weeks
- Once per month
- Once every two months
- Once per quarter (every three months)
- Once per year
- Less than once per year
- Never

Table A1 — Analysis of Sociodemographic Factors

Dependent Variable
Propensity too btain pre Number of phar- Duration per visit
scription medicine online wt : 7
. macy visits (in min)
(in sd)
ic) (2) (3)

Germany -0.705*** 1.067 -5.146**
(0.0887) (2.026) (2.530)

Spain -0.568*** 10.80*** -6.720***
(0.0437) (0.846) (1.134)

France -0.845*** 2.951*** -5.571***
(0.0430) (0.680) (1.107)
Italy -0.189*** 12.26%** -1.219
(0.0452) (0.945) (1.268)

Age 18-44 (relative to 65+) 0.407*** 3.173%** 14.55***
(0.0382) (0.755) (1.053)

Age 45-64 (relative to 65+) 0.276*** 0.554 3.620***
(0.0389) (0.676) (0.854)
Female 0.0615** -0.0619 -0.798
(0.0302) (0.635) (0.841)

Income (in EUR 10,000) 0.0139** -0.208 -0.541**
(0.00675) (0.170) (0.214)
Frequent Online Shopper 0.404*** 7.28228* 1.454*
(0.0312) (0.6901) (0.882)
Level of Education 0.0191* -0.286 -0.166
(0.00992) (0.212) (0.275)

City Size < 1,000 -0.0480 0.301 7.047***
(0.0632) (1.111) (1.764)

Constant -0.0905 8.596*** 27.64***
(0.0653) (1.275) (1.719)

Observations 3820 3829 3820

R2 0.190 0.118 0.076

Note: The table shows the regression results from three linear regression models: Model 1 shows the effect of sociodemographic factors on the propensity to obtain prescription medicine online. Model 2 shows the effect of sociodemographic factors on the number of pharmacy visits and model 3 shows the effect of sociodemographic factors on the duration per visit. The country dummy variables show county-fixed effects relative to the effect observed for Sweden. The two age groups show the effect of the two age groups relative to the age group of people older than 65 years. Female shows the effect of women relative to individuals who do not identify as women. The income variable is continuous and shows the effect of different income levels. Frequent Online Shopper is a binary variable and captures the effect of individuals who indicate making more than 12 online purchases per year. Note that this is not limited to pharmacy products but to all online purchases. The level of education is continuous and captures various levels of education. Finally, City Size is a binary variable capturing the effect of living in a city with less than 1,000 inhabitants relative to cities with a larger population. For all models, we use robust standard errors. *, ** and *** mark significance levels of p<.05, p<.01 and p<.001, respectively.
